# Genome Sequence Analysis Reveals Selection Signatures in Endangered Trypanotolerant West African Muturu Cattle

**DOI:** 10.3389/fgene.2019.00442

**Published:** 2019-06-04

**Authors:** Abdulfatai Tijjani, Yuri Tani Utsunomiya, Arinze G. Ezekwe, Oyekanmi Nashiru, Olivier Hanotte

**Affiliations:** ^1^Cells, Organisms and Molecular Genetics, School of Life Sciences, University Park Campus, University of Nottingham, Nottingham, United Kingdom; ^2^Center for Genomics Research and Innovation, National Biotechnology Development Agency, Abuja, Nigeria; ^3^International Livestock Research Institute, Addis Ababa, Ethiopia; ^4^Department of Support, Production and Animal Health, School of Veterinary Medicine, São Paulo State University, São Paulo, Brazil; ^5^Department of Animal Science, Faculty of Agriculture, University of Nigeria, Nsukka, Nigeria

**Keywords:** African *Bos taurus*, MHC, disease resistance, heat tolerance, comparative genomics

## Abstract

Like most West African *Bos taurus*, the shorthorn Muturu is under threat of replacement or crossbreeding with zebu. Their populations are now reduced to a few hundred breeding individuals and they are considered endangered. So far, the genetic variation and genetic basis of the trypanotolerant Muturu environmental adaptation have not been assessed. Here, we present genome-wide candidate positive selection signatures in Muturu following within-population *iHS* and between population *Rsb* signatures of selection analysis. We compared the results in Muturu with the ones obtained in N’Dama, a West African longhorn trypanotolerant taurine, and in two European taurine (Holstein and Jersey). The results reveal candidate signatures of selection regions in Muturu including genes linked to the innate (e.g., *TRIM10, TRIM15, TRIM40*, and *TRIM26*) and the adaptive (e.g., *JSP.1, BOLA-DQA2, BOLA-DQA5, BOLA-DRB3*, and *BLA-DQB*) immune responses. The most significant regions are identified on BTA 23 at the bovine major histocompatibility complex (MHC) (*iHS* analysis) and on BTA 12 at genes including a heat tolerance gene (*INTS6*) (*Rsb* analysis). Other candidate selected regions include genes related to growth traits/stature (e.g., *GHR* and *GHRHR*), coat color (e.g., *MITF* and *KIT*), feed efficiency (e.g., *ZRANB3* and *MAP3K5*) and reproduction (e.g., *RFX2, SRY, LAP3*, and *GPX5*). Genes under common signatures of selection regions with N’Dama, including for adaptive immunity and heat tolerance, suggest shared mechanisms of adaptation to environmental challenges for these two West African taurine cattle. Interestingly, out of the 242,910 SNPs identified within the candidate selected regions in Muturu, 917 are missense SNPs (0.4%), with an unequal distribution across 273 genes. Fifteen genes including *RBBP8, NID1, TEX15, LAMA3, TRIM40*, and *OR12D3* comprise 220 missense variants, each between 11 and 32. Our results, while providing insights into the candidate genes under selection in Muturu, are paving the way to the identification of genes and their polymorphisms linked to the unique tropical adaptive traits of the West Africa taurine, including trypanotolerance.

## Introduction

The Muturu cattle, indigenous to the West African sub-region, is among the least characterized shorthorn (brachyceros) humpless *Bos taurus*, which include also the Somba, Baoulé, and Lagune. The word Muturu, the Hausa word for humpless, is used for all taurine shorthorns in English-speaking West African countries. The origin of the Muturu is disputed, their ancestors are likely the shorthorn humpless cattle which appeared in ancient Egypt in the second millennium BC, migrating from the center(s) of cattle domestication in the Near East. They were first recorded in West Africa during the first half of the first millennium AD (Epstein, 1971). The past distribution of Muturu ranged across West and Central Africa, particularly in Cameroon, Liberia, Ghana and Nigeria. Nowadays, Muturu is sparsely distributed in the humid forest zone and in a few savannah areas in Nigeria (Rege et al., 1994; Adebambo, 2001; Gwaza and Momoh, 2016).

Two Muturu ecotypes may be recognized in Nigeria. The Savannah Muturu, the larger one, found in pockets of central North (Benue plateau and surroundings) and in the Eastern parts of the country, and the Dwarf Forest Muturu in South West Nigeria and along the coastline bordering Benin and Cameroun. It is thought that Muturu populated most of Nigeria before the arrival of zebu *B. indicus* cattle in the 17th century (Felius, 1995). Since then, the existence of Muturu has been consistently under threat as a result of farmers’ preference for zebu larger body size. Also, the late 19th century cattle rinderpest plagues seem to have caused the extinction of many Muturu herds (Blench, 1998). Following its rapid decline, from 115,000 cattle heads reported in 1992 to a recent unofficial estimate of about 25,000, the Muturu has now been classified in Nigeria as an endangered breed (Adebambo, 2001).

Muturu are the smallest cattle of Nigeria and also the smallest among the shorthorns in West Africa, with reported wither heights varying from 71 to 100 cm (Maule, 1990; Rege et al., 1994). Muturu are important in traditional culture and there is a strong spiritual attachment to the animals. They are frequently used for prestige and dowry purposes. The animals play an important role in all kinds of ceremonies and they are used for ritual sacrifices, particularly at funerals, when their hides are used as wrapping for the deceased. Many bulls are therefore killed for such purposes which may lead to shortage of breeding bulls (Blench, 1998). Muturu cattle have good temperament and can be highly productive when managed under station condition. They are known for their tolerance to the parasitic protozoal disease, trypanosomiasis, due to their innate ability to remain productive even after infection (Murray, 1988). They are also tolerant to tick and tick-borne diseases, but they are susceptible to rinderpest (Rege et al., 1994).

The genetic variation and loci under selection across the genome of Muturu have not been so far assessed. Such investigations are of relevance for conservation purposes (e.g., inbreeding avoidance), breed management and for the understanding of the genetic bases of local adaptive traits. In this study, we report genome-wide assessment of candidate positive selection signatures in a Muturu population from Nigeria using whole genome re-sequencing data. These analyses involved the assessment of genetic diversity, population structure and signatures of positive selection, using two complementary extended haplotypes homozygosity selection tests, within population, integrated haplotype score (*iHS*) and between population, *Rsb*. Muturu results were compared to N’Dama, a West African longhorn trypanotolerant taurine, and two European taurine breeds (Holstein and Jersey).

## Materials and Methods

### Study Populations and Whole Genome Re-sequencing

The genome sequences of the Muturu samples included in this study were recently made available in the public domain following the inclusion of these samples as reference population in a study of adaptation of the East African shorthorn zebu (Bahbahani et al., 2017). Sequenced reads are available in NCBI SRA database under the project accession: PRJNA386202. Tissue samples were collected from the earlobes of 10 Muturu animals, which were randomly sampled in villages in Nsukka (6.830801″ N, 7.413971″ E) and Igbo-Etiti (6.692760″ N, 7.398349″ E), Local Government Areas in Southeast Nigeria. Total genomic DNA was extracted following the manufacturer’s protocol using the Macherey-Nagel NucleoSpin^®^ tissue DNA extraction kit. Samples were sent to a commercial company (Novogene) for sequencing, where paired-end libraries with an insert size of 150 bp were constructed from at least 5 μg genomic DNA according to the Illumina’s library preparation protocol. Paired-end sequencing of libraries was performed on the Illumina Hiseq 2500 platform (Illumina Inc., San Diego, CA, United States), samples were sequenced to about 10x coverage. The genome sequence data of 10 cattle samples each from three other *B. taurus* populations (N’Dama, Holstein and Jersey) were obtained from previous studies (accessions numbers PRJNA312138, PRJNA176557 and PRJNA238491, respectively) (Daetwyler et al., 2014; Stothard et al., 2015; Kim et al., 2017).

### Sequence Mapping and Variants Discovery

The generated raw sequence data were subjected to filtering steps in order to remove reads pair containing adapter and when more than half of the reads comprise of low base quality (Q_phred_ < 5). The final quality of the resulting clean reads was confirmed using the fastqc tool^1^. Clean sequence reads for the individual cattle samples were aligned to the *B. taurus* reference genome (UMD 3.1) (Zimin et al., 2009) using the Burrows-Wheeler Alignment tool (BWA) version 0.7.5a with the option “bwa-mem” (Li and Durbin, 2009). We followed the Broad Institute recommended Genome Analysis Toolkit (GATK) Best Practices pre-processing workflow preceding variant discovery^2^. We used SAMtools v1.1 (Li et al., 2009) to convert SAM files to BAM files. Picard tools v1.119^3^ was used to sort the alignment files by coordinates, to index them, to mark duplicate reads and to generate quality matrices after mapping. We then performed local re-alignment around InDels and recalibration of the base quality scores with GATK *v3.4* (McKenna et al., 2010), using the default settings and the dbSNP Build 148^4^ as list of known variant sites.

Discoveries of single nucleotide variants (SNVs) and Insertion/Deletions (InDels) on individual cattle sample was performed using the default parameters of the GATK “*HaplotypeCaller*.” Thereafter, the joint genotyping approach (*GenotypeGVCFs* mode) was adopted to identify variants simultaneously in all *B. taurus* samples. GATK “*SelectVariants*” mode was used to separate SNPs and InDels into two different files before subjecting each variant type to hard filtering in order to reduce false positive variants. We excluded SNPs with “DP < 4” (SNPs covered by less than 4 reads), “MQ < 40.0” (SNPs with low mapping quality), quality by depth, QD < 2.0 and other GATK recommended default SNP filtering criteria; “MQRankSum < -12.5,” “ReadPosRankSum < -8.0,” and Fisher strand, “FS > 200.” The InDels were filtered with the following criteria; QD < 2.0, FS > 200.0 and ReadPosRankSum < -20.0. BCFtools *v* 0.1.14 was used to estimate SNP statistics such as the numbers of total and average bi-allelic autosomal SNPs, ratios of heterozygous to homozygous SNPs and ratios of transition to transversion (Ts/Tv) in each *B. taurus* breeds.

### Genetic Relationship and Population Structure Analyses

For genetic relationship analysis, VCFtools *v.*0.1.14 (Danecek et al., 2011) was used to select SNPs with 100% genotyping rate across the four *B. taurus* populations which were then subjected to a further thinning by selecting a single SNP per 1000-kb window resulting in a dataset of about 1.9 million SNPs. These SNPs were loaded into the R software using the vcfR package (Knaus and Grünwald, 2017) to create a vcfR object and subsequently converted into a genlight object (Jombart, 2008). Using this package, we estimated pairwise Nei’s Genetic Distances (*D*) (Nei, 1972) between populations and between individuals. A heat map was plotted within the R environment to visualize the relationship between African and European *B. taurus* based on Nei’s Genetic Distances.

Principal component (PCA) and admixture analyses were performed to infer the population structure among the four *B. taurus* cattle. VCFtools *v.0.1.14* was used to convert the entire genotype dataset into Plink input file format. PCA was then performed using the SmartPCA software from the EIGENSOFT (Patterson et al., 2006) package, and the first four eigenvectors were plotted. Block relaxation algorithm implemented in the ADMIXTURE *v1.3.0* program (Alexander et al., 2009) was used for analysis of ancestry proportions (admixture) with K set from 2 to 4. The whole genotype data set was subjected to linkage disequilibrium (LD) pruning using the default parameter of PLINK *v1.9* (50 SNPs step 5 SNPs, r2 0.5) (Purcell et al., 2007) prior to use in admixture analysis. GENESIS software (Buchmann and Hazelhurst, 2014) was used to visualized both PCA and admixture plots.

### Signature of Selection Using Integrated Haplotype Score (iHS) Test

The *iHS* statistical test is a haplotype-based signature of selection scan method that is computed based on the estimation of extended haplotype homozygosity (EHH) from each core bi-allelic SNPs, which are either ancestral or derived. In this case, with the use of whole genome re-sequencing data aligned to the bovine reference genome (UMD3.1), the reference allele is considered the ancestral while that of our study population is considered the derived allele. The sum of EHH computed over both directions away from the core SNP is referred to as integrated EHH. It is denoted iHHA for the ancestral allele, iHHD for derived allele and iES for the SNP site (Qanbari et al., 2011; Gouveia et al., 2014). *iHS* is based on the extent of decay of LD at a variant, it is thus described as a within population score as the ratio between iHHA and iHHD:

(1) $$iHS=\ln\left(\frac{iHHA}{iHHD}\right)\;\;\;\;\;\;\;\;\;\;\;\;(\mathrm{Voight}\;\mathrm{et}\;\mathrm{al}.,\;2006)$$

The *REHH* package (Gautier and Vitalis, 2012) in R was used to compute *iHS* score for SNPs with MAF ≥ 0.05 within each of the four *B. taurus* populations separately and using the option “*freqbin* = *0.1*.” The *freqbin* option enables the calculation of empirical *P*-values for every score based on the number of SNPs within a bin of similar allele frequency of size 0.1 (Voight et al., 2006). *piHS* were derived as -log10(1-2|Φ(iHS)-0.5|), where, Φ(iHS) represents the cumulative distribution function of the Gaussian density (Gautier and Vitalis, 2012).

To infer candidate regions under positive selection, we utilized an in-house R script to summarize the *iHS* scores for each SNP within a sliding window of 100 kb with an overlap of 50 kb step, selecting SNPs with the highest test score to represent a particular window. For any window to be considered as significant, we applied a threshold of *P* < 0.0001, equivalent to *piHS* > 4, corresponding to false discovery rates (FDR) of 0.0084, 0.0080. 0.0041 and 0.0128 in Muturu, Holstein, N’Dama and Jersey, respectively, when applying the approach of Storey and Tibshirani (2003). Bedtools *v2.25.0* (Quinlan and Hall, 2010) was then used to merge overlapping significant windows to define a candidate selected region.

As this test require phased haplotypes (Sabeti et al., 2002; Voight et al., 2006), phasing was performed separately for each *B. taurus* population and for each chromosome using the SHAPEIT software (Delaneau et al., 2013). We adopted the read aware phasing approach which takes advantage of the phase information contained in sequencing reads to improve the quality of the resulting haplotypes. Following this approach, we extracted Phase Informative Read (PIR, which is defined as a sequencing read that span at least 2 heterozygous sites), from the sequence alignment files (Bam files) for all samples and then performed the actual phasing of the genotypes contained in the variant file using the extracted PIRs as an additional input to SHAPEIT (Delaneau et al., 2013).

### Signature of Selection Using Extended Haplotype Homozygosity, *Rsb* Test

Evidence of positive selection in Muturu was further investigated by comparing Muturu to each of the other three *B. taurus* breeds using the *Rsb* test, which is also based on the estimation of EHH as described above. However, in contrast to *iHS*, where EHH is compared between alleles at a SNP within a population, *Rsb* involved the comparison of the EHH patterns of the same allele (denoted as “iES”) between two populations. Tang et al. (2007), described *Rsb* as:

(2) $$\ln(Rsb)=\ln\left(\frac{iESpop1}{iESpop2}\right)\;\;\;\;\;\;\;\;\;\;\;\;(\mathrm{Tang}\;\mathrm{et}\;\mathrm{al}.,\;2007)$$

To obtain *Rsb* scores, we utilized the different iES statistics estimated using REHH package during the *iHS* analyses in each of Muturu, N’Dama, Holstein and Jersey populations as described above. The iES values were summarized using overlapping window size of 100 kb with overlapping step 50 kb as for *iHS* analysis. *Rsb* scores were then computed by comparing Muturu with N’Dama, Holstein and Jersey using Equation 2. *Rsb* scores were standardized in R environment. As *Rsb* score is directional, a positive *Rsb* score suggests selection in population 1 while the negative score suggests selection in population 2. In our case, selection signature in Muturu is represented by the positive values. To select candidate selection signature windows, the 0.5% extreme values were considered. Bedtools *v2.25.0* (Quinlan and Hall, 2010) was used to merge overlapping selected windows as selected region.

### Functional Characterization of Candidate Selected Genes and Haplotype Diversity

Cattle genes based on Ensembl Genes 92 database, which overlapped the genomic coordinates (in bp) of our significant selective sweep regions were retrieved using *Ensembl BioMart* online tool^5^ (last accessed on June 10, 2018) (Smedley et al., 2009). The list of the retrieved genes was processed in the web-based Database for Annotation, Visualization and Integrated Discovery (DAVID) v6.8^6^ tool (Huang et al., 2009a,b) for functional annotation and identification of enriched terms including Gene ontology (GO) biological processes and Kyoto Encyclopedia of Genes and Genomes (KEGG) pathways, associated with out gene list. For DAVID analysis, the modified Fisher exact test *P*-value was used to estimate the significance of each functional annotation term.

Further characterization of candidate selected genes was performed by comparing the missense variants within candidate selected regions among the four *B. taurus* breeds. Furthermore, haplotype diversity in regions of candidate genes was investigated by using the scripts provided in the hapFLK webpage^7^ for the estimation of haplotype frequencies and visualization of haplotype clusters within selected candidate genes (Fariello et al., 2013).

## Results

### Variants Discovery and Statistics

Sequencing of the 10 Muturu samples generated a total of 324.179 Gigabase pairs (Gbp) of clean sequence data after trimming of adapters and low-quality reads. Individual sample sequence reads ranged from 188,884,710 to 240,635,522. Mapping of each sample sequence reads to the *B. taurus* cattle genome of reference UMD 3.1, yielded a minimum alignment rate of 0.99 and an assembly coverage of 98 percent in all 10 Muturu samples. The genome coverage for each Muturu sample ranged from 6.45 to 10.41 with a total of 83.28 folds genome coverage (Supplementary Table S1). The sequences statistics for the genomes of Jersey, Holstein and N’Dama populations are provided in Daetwyler et al. (2014), Stothard et al. (2015), Kim et al. (2017), respectively. The genomes coverage of individual animal is around 10 folds for these three breeds.

**FIGURE 1:**
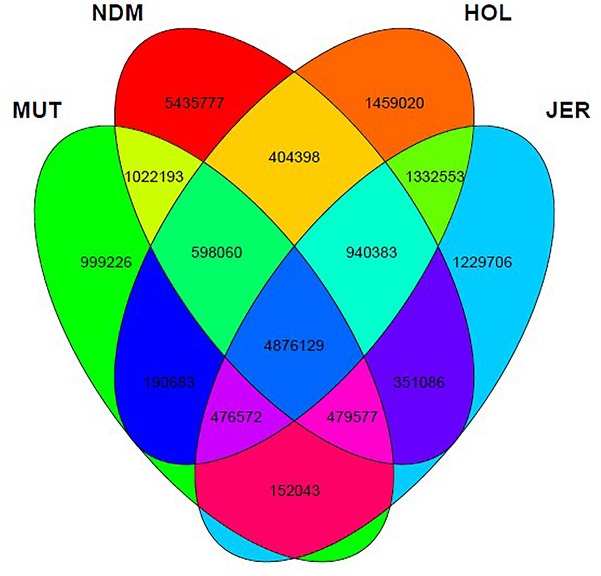
Venn diagram of unique and shared autosomal bi-allelic SNPs between Muturu and other taurine. MUT, Muturu; NDM, N’Dama; HOL, Holstein; JER, Jersey.

After the SNPs quality control steps, 8,794,483, 14,107,603, 10,277,798, and 9,838,049 autosomal bi-allelic SNPs were obtained in Muturu, N’Dama, Holstein and Jersey, respectively (Table 1), giving a total of 19,947,406 SNPs (shared and breed-specific SNPs). Amongst these, 4,876,129 SNPs are common to all four populations, a proportion equivalent to 55.4, 49.6, 47.4, and 34.6% of autosomal SNPs in Muturu, Jersey, Holstein and N’Dama, respectively; 1,022,193 (11.6%) are common to Muturu and N’Dama, 999,226 (5%) are unique to Muturu and 5,435,777 (27%) unique to N’Dama (Figure 1). So more than 43% of the SNPs are unique to African cattle compared to the two European breeds. More so, new SNPs are higher in the African *B. taurus* (∼5%) than in the European counterpart (less than 1%) following comparison with the cattle SNPs database dbSNP Build 148^4^.

**Table 1:** Summary statistics of the SNPs in the four Bos taurus cattle.

SNP stats	Muturu	N’Dama	Holstein	Jersey
Total bi-allelic autosomal SNPs	8,794,483	14,107,603	10,277,798	9,838,049
Average number of SNPs per sample	4,841,893	7,116,855	5,180,989	4,650,501
Het/Hom	0.69	1.53	1.56	1.16
Ts/Tv ratio	2.29	2.31	2.28	2.28
Singleton SNPs (%)	13.9	12.7	16.3	16.9
dbSNP (%)	94.7	95	99.6	99.5


The average transition to transversion (Ts/Tv) ratio is quite similar across breed, 2.28 in European taurine, 2.29 in Muturu and 2.31 in N’Dama (Table 1). This number is quite comparable to what has been reported in other cattle studies (Choi et al., 2015; Stothard et al., 2015; Stafuzza et al., 2017). It is indicative of high quality of our variant call sets. Among the different *B. taurus* breeds, the ratio of heterozygous to homozygous non-reference SNPs is the lowest in the Muturu breed (het/hom = 0.69), while the ratios are above 1.0 in other breeds. The numbers of singletons SNPs are lower in the two West African taurine than in the two European taurine (Table 1).

### Genetic Relationships and Within Population Structure

**FIGURE 2:**
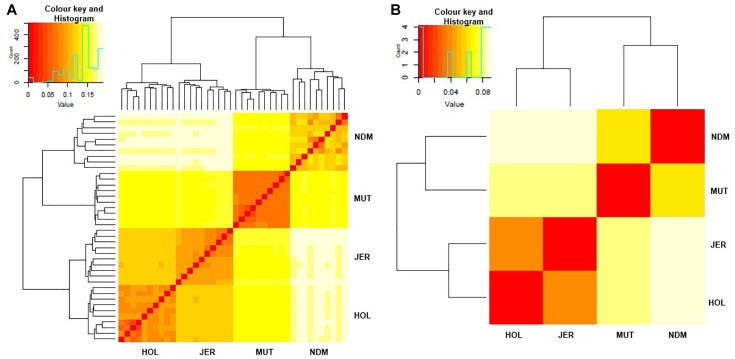
Heat map of genetic relationships among the four *B. taurus* based on average Nei’s genetic distances matrix. **(A)** individuals and **(B)** populations comparisons. MUT, Muturu; NDM, N’Dama; HOL, Holstein; JER, Jersey.

We examined genetic relationships among the animals and populations by computing Nei’s genetic distances (*D*) (Nei, 1972; Supplementary Tables S2, S3). The relationships among individuals and population are presented as heat maps at Figure 2A,B. From the individual sample plot (Figure 2A), samples of the same population clearly cluster together. Samples within the Muturu population are more related to each other compared to the N’Dama samples as indicated by the corresponding dark and light colors of the squares (Figure 2A). Within N’Dama, some genetic distance heterogeneities among animals are observed in relation to the European taurine Holstein and Jersey. At population level (Figure 2B), the genetic distances heat map shows a closer genetic relationship between the two European breed (*D* = 0.041) than between the two African (*D* = 0.066) populations. The highest genetic distance is observed between N’Dama and Jersey (*D* = 0.089). Muturu has a closer relationship with the two European taurine than N’Dama.

**FIGURE 3:**
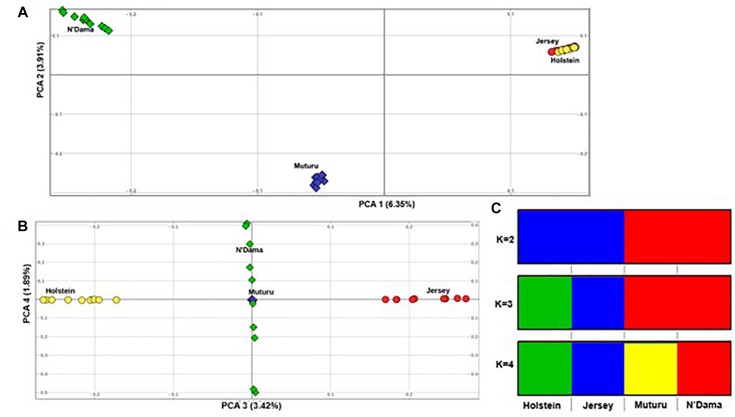
Population structure of the four *B. taurus* breeds. **(A)** PC 1 versus PC 2, **(B)** PC 3 versus PC 4 and **(C)** Admixture analysis *K* = 2–4.

Principal component analysis supports the population relationships based on average genetic distances. The plot of the first (PC1) and second eigenvectors (PC2) (Figure 3A), explaining about 6.4 and 3.5% of the proportion of variations, respectively, shows a clear separation of the African taurine from the European taurine, and a separation between the two African taurine, respectively. PC3 separates the two European taurine while PC4 reveals within breed diversity in N’Dama (Figure 3B). The admixture level indicates clustering at continental level at *K* = 2. At *K* = 3, the European populations are separated, while the African populations are still clustered together. A separate ancestry for all four populations is visible at *K* = 4 (Figure 3C).

### Candidate Selection Signatures of Muturu as Revealed by *iHS*

**FIGURE 4:**
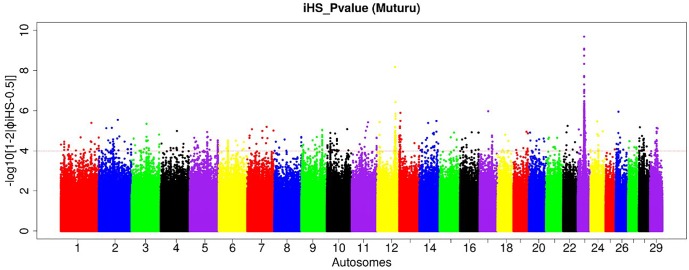
Manhattan plot of the genome-wide distribution of *iHS* scores for the autosomes in Muturu cattle. The red dash line indicates the threshold of *P* < 0.0001 [equivalent of –log (*iHS*) > 4] at which windows are considered under selection.

To investigate ongoing within breed recent selection in Muturu, we used the EHH-based *iHS* test. Genome-wide within population *iHS* scores was calculated using all SNPs with a minimum allele frequency of 5% in the breed. The distributions of the standardized *iHS P*-values along the 29 bovine autosomes are shown at Figure 4. Using a threshold of *iHS* ≥ 4 (*P*-value < 0.0001), we identified 266 significant genomic regions under positive selection in Muturu (Supplementary Table S4). From these, 169 regions overlap with 430 genes, while the remaining 97 regions are gene desert regions according to the *Ensembl* cow genes database 92.

Amongst the top 10 candidate selection signature regions, beside several known bovine genes, we do find also several others yet to be annotated (Table 2). The strongest selection signal in Muturu is observed on BTA 23 [-log10 (p*iHS*) = 9.69] (Figure 4), and overlapping an unannotated protein coding gene, *ENSBTAG00000026163*. Here, among the functionally annotated genes, we do find the bovine major histocompatibility complex (MHC) class I (*JSP.1, BOLA*) and class II (*BLA-DQB*), the olfactory receptor genes family (*OR5V1* and *OR12D3*), tripartite motif-containing (TRIM) genes (*TRIM10, TRIM15, TRIM26*, and *TRIM40*), and the *NOTCH4, PBX2, RNF5, PPT2, PRRT1*, and *AGER* genes. Other top selection signatures regions based on *iHS* analysis were detected on chromosomes BTA 12, 13, 17, and 26 (Table 2).

**Table 2:** Most significant 10 *iHS* candidate selection signature regions in Muturu.

Chr	Start (bp)	End (bp)	-log10 (*iHS*)	Genes overlapping candidate selected region^∗^
23	26200001	26400000	9.69	*ENSBTAG00000026163*
12	73950001	74300000	8.17	*ENSBTAG00000047360, ENSBTAG00000047764*
23	26650001	27050000	7.03	*ENSBTAG00000046920, PBX2, AGER, RNF5, EGFL8, PRRT1, ENSBTAG00000046116, ENSBTAG00000023563, ENSBTAG00000048304, NOTCH4, AGPAT1, PPT2, ENSBTAG00000023541*
23	25700001	26200000	6.02	*BLA-DQB, ENSBTAG00000033979*
17	35800001	35950000	5.97	*ENSBTAG00000045738, ENSBTAG00000045938*
26	11450001	11600000	5.95	*KIF20B*
13	5300001	5450000	5.89	*SRY*
23	28400001	28650000	5.66	*JSP.1, BOLA, ENSBTAG00000037421, ENSBTAG00000044550, TRIM10, TRIM40, TRIM15, TRIM26*
23	29150001	29400000	5.50	*OR12D3, ENSBTAG00000039901, OR5V1, ENSBTAG00000013654, ENSBTAG00000031843, ENSBTAG00000039534, ENSBTAG00000046777, ENSBTAG00000047086, ENSBTAG00000046023, ENSBTAG00000038608, ENSBTAG00000037628, ENSBTAG00000031850, ENSBTAG00000000228*
14	71350001	71500000	5.49	*C8orf37*


*^∗^As per cow genes 92 *Ensembl* database.*

### Candidate Selection Signatures of Muturu as Revealed by *Rsb*

**FIGURE 5:**
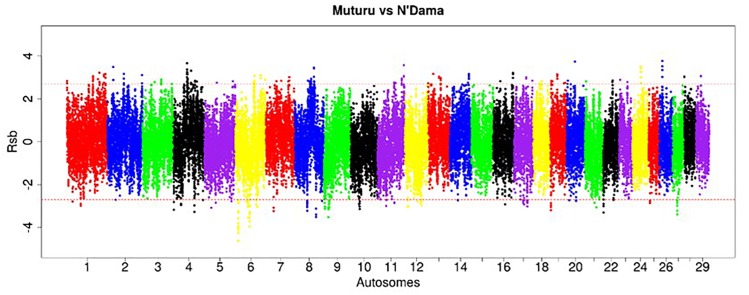
Manhattan plot of the genome-wide distribution of *Rsb* Muturu – N’Dama. Red dash lines are the threshold of the windows in the top 0.5% *Rsb* scores.

**FIGURE 6:**
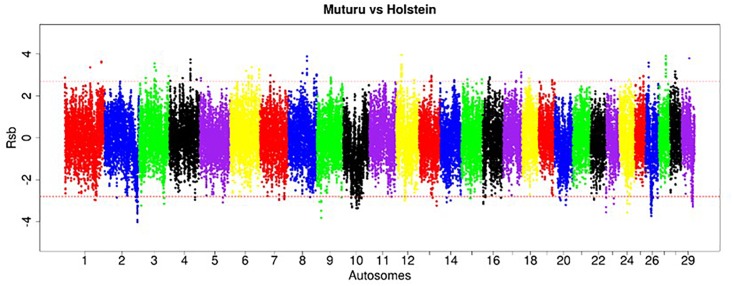
Manhattan plot of the genome-wide distribution of *Rsb* Muturu – Holstein. Red dash lines are the thresholds of the windows in the top 0.5% *Rsb* scores.

**FIGURE 7:**
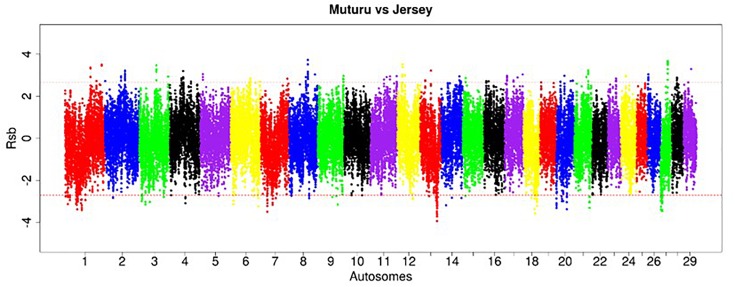
Manhattan plot of the distribution of *Rsb* Muturu – Jersey. Red dash lines are the thresholds of the windows in the top 0.5% *Rsb* scores.

The EHH-based *Rsb* approach was used to unravel the genomic signatures of selection in Muturu by comparing its genome to each of three other taurine breeds (N’Dama, Holstein and Jersey). The distribution of *Rsb* values along bovine autosomes are presented in the Manhattan plots shown in Figure 5–7. A total of 145 selection signature regions is identified, out of which 81 overlap with 206 candidate genes with the remaining 64 being gene desert regions (Supplementary Table S5).

**FIGURE 8:**
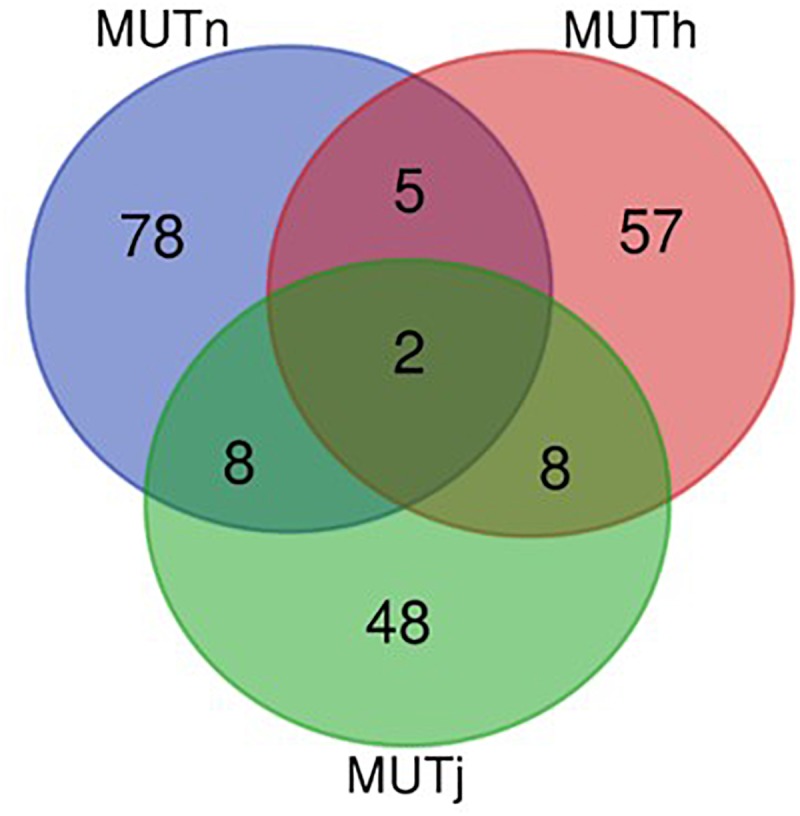
Venn diagram of unique and shared genes within candidate selected regions between Muturu and other taurine following *Rsb* analysis. MUTn, Muturu – N’Dama; MUTh, Muturu – Holstein; MUTj, Muturu – Jersey.

Ninety-three, 73 and 66 genes were identified following the comparison of Muturu with N’Dama, Holstein and Jersey, respectively (Figure 8). Overlaps among the three sets show two genes (*TFEC* and *OSBP2*) detected in the three comparison tests, and twenty-one in two different comparison tests (Figure 8). Among these, eight genes including *FAM124A, PIGK, ST6GALNAC5, WDR49, SERPINI2, CD38, ENSBTAG00000033558*, and *ENSBTAG00000047597* were present in regions commonly identified between the Muturu and the two European taurine breeds. These 23 candidate genes are found within candidate selected regions on chromosomes 1, 3, 4, 6, 8, 12, 13, and 17 (Table 3).

**Table 3:** List of overlapping candidate genes detected by *Rsb* analysis following the comparison of Muturu with each of three other taurine breeds (N’Dama, Holstein and Jersey).

Chr	Start (bp)	End (bp)	Gene	*Rsb* comparisons
1	351708	362907	*RCAN1*	N’Dama, Holstein
1	100676575	100786489	*WDR49*	Holstein, Jersey
1	100791772	100827942	*SERPINI2*	Holstein, Jersey
3	67687802	67824632	*PIGK*	Holstein, Jersey
3	67863093	68073051	*ST6GALNAC5*	Holstein, Jersey
4	52589574	52812807	*TFEC*	N’Dama, Holstein, Jersey
4	52989410	52989499	*ENSBTAG00000045017*	N’Dama, Jersey
6	115802869	115858942	*CD38*	Holstein, Jersey
6	115940219	115943190	*FGFBP1*	N’Dama, Holstein
6	115981183	116105180	*PROM1*	N’Dama, Holstein
8	74834628	74913332	*PPP2R2A*	N’Dama, Jersey
8	74924184	74947549	*BNIP3L*	N’Dama, Jersey
8	75089346	75168436	*DPYSL2*	N’Dama, Jersey
8	75108590	75108708	*ENSBTAG00000029080*	N’Dama, Jersey
12	20704476	20778873	*FAM124A*	Holstein, Jersey
13	42398735	42399402	*NXT1*	N’Dama, Jersey
13	42407301	42413062	*GZF1*	N’Dama, Jersey
13	42416038	42453614	*NAPB*	N’Dama, Jersey
17	51217469	51219592	*ENSBTAG00000047597*	Holstein, Jersey
17	51260767	51262423	*ENSBTAG00000033558*	Holstein, Jersey
17	71750227	71755793	*DUSP18*	N’Dama, Holstein
17	71755943	71760583	*C17H5orf52*	N’Dama, Holstein
17	71771703	71894872	*OSBP2*	N’Dama, Holstein, Jersey


The strongest candidate selective sweep region in Muturu is on BTA 12 (550 Mb region; position 20550001 – 21100000, *Rsb* = 3.95) (Table 4). It is detected following *Rsb* analysis between Muturu and the European taurine breeds. The candidate genes present here are *FAM124A, SERPINE3, WDFY2*, and *INTS6* (Table 4). *FAM124A* was detected following comparison of Muturu with Holstein and Jersey, while the remaining three genes (*SERPINE3, WDFY2*, and *INTS6*) were detected following the comparison with Holstein only. Other identified strong selection signatures regions and candidate genes in Muturu based on the 15 top *Rsb* scores are presented at Table 3. Candidate selected regions identified on chromosomes BTA 2 (*OLA1*), BTA 11 (*GFI1B, SPACA9, GTF3C5, CEL, RALGDS, AK8*, and *TSC1*), BTA 24 (*ANKRD29, NPC1, RMC1*, and *LAMA3*), and BTA 26 (*KIF20B*) are the results of the *Rsb* analysis between Muturu and N’Dama. These genes may, therefore, provide insights into the genetic adaptive differences between the shorthorn and longhorn taurine of West Africa.

**Table 4:** Most significant 15 *Rsb* selection signatures regions in Muturu.

Chr	Start (bp)	End (bp)	*Rsb*	Genes overlapping the candidate selected regions
12	20550001	21100000	3.95	*SERPINE3, FAM124A, WDFY2, INTS6*
27	28450001	29000000	3.90	*RNF122, DUSP26*
26	11450001	11800000	3.77	*KIF20B*
4	52650001	53100000	3.66	*TFEC*
11	102900001	103150000	3.56	*GFI1B, SPACA9, GTF3C5, CEL, RALGDS, AK8, TSC1*
24	33250001	33500000	3.52	*ANKRD29, NPC1, RMC1, LAMA3*
2	22650001	22800000	3.49	*OLA1*
3	67600001	68000000	3.47	*AK5, PIGK, ST6GALNAC5*
8	74850001	75300000	3.46	*PNMA2, BNIP3L, ADRA1A, PPP2R2A, DPYSL2*
6	86650001	86900000	3.37	*UGT2A1*
1	100750001	100900000	3.36	*WDR49, SERPINI2, ZBBX*
1	101000001	101200000	3.33	*ZBBX*
4	68650001	68750000	3.31	*JAZF1*
6	115750001	116050000	3.27	*FGFBP1, CD38, PROM1*
21	54650001	54900000	3.23	*FSCB*


### Comparison of Selection Signatures in Muturu and Other Taurine *Breeds*

To enable us to compare the selection signatures in Muturu with the ones in the other *B. taurus* breeds (N’Dama, Holstein and Jersey), we additionally performed selection scans of the genomes of each of the three other taurine breeds both within population (*iHS*) and between them (*Rsb*). The distributions of these genome-wide *iHS* and *Rsb* scores are presented in Supplementary Figures S1–S6.

**FIGURE 9:**
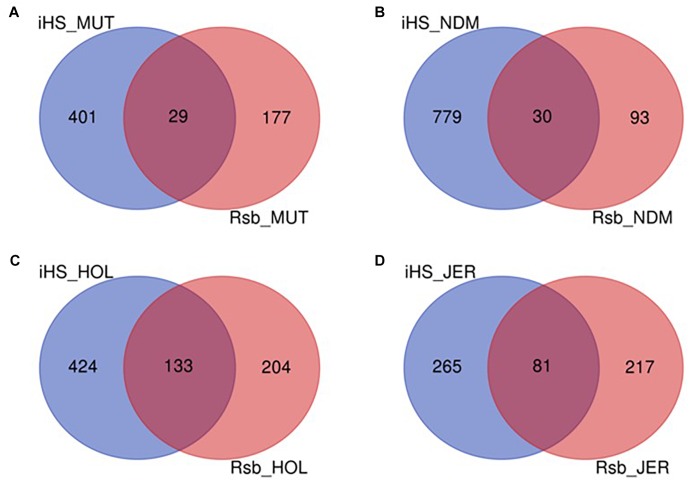
Venn diagram showing the unique and shared candidate genes in the four *B. taurus* following *iHS* and *Rsb* analysis. **(A)** Muturu (MUT), **(B)** N’Dama (NDM), **(C)** Holstein (HOL), **(D)** Jersey (JER).

The numbers of candidate genes identified in N’Dama, Holstein and Jersey based on the *iHS* test are 809, 557, and 346, respectively (Supplementary Tables S6–S8). The numbers of genes detected following the *Rsb* analysis of N’Dama, Holstein and Jersey are 123, 337, and 298, respectively (Supplementary Tables S9–S11). Figure 9 summarizes the number of candidate genes detected by both tests and their overlaps for each of the four breeds. Nine hundred and two, 761 and 563 candidate genes excluding duplicates, were identified in N’Dama, Holstein and Jersey. This total is 607 in Muturu. The numbers of detected genes associated with fixed or close to fixation selective sweeps (*Rsb*) are higher in the European breeds than in Muturu and N’Dama (Figure 9). Likewise, higher numbers of overlapped candidate genes between the two selection scan tests are observed in the European taurine than in the West African taurine (Figure 9).

**FIGURE 10:**
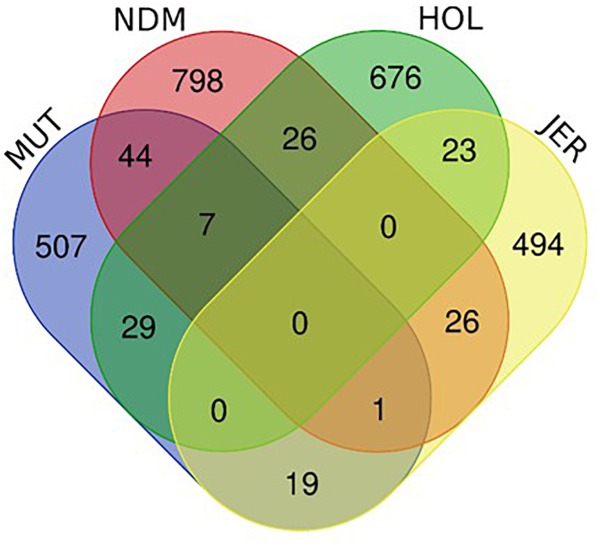
Venn diagram showing unique and shared candidate genes identified in candidate regions under selection (*iHS* and *Rsb*) in Muturu (MUT), N’Dama (NDM), Holstein (HOL), and Jersey (JER).

The numbers of unique and shared candidate genes for the four taurine breeds is shown in Figure 10. Comparisons of the candidate genes identified in each of the taurine breeds indicate a moderate overlap between pairs of taurine breeds, with no candidate gene common to the four breeds (Figure 10). More precisely, the numbers of genes within selected regions in Muturu shared with N’Dama, Holstein and Jersey are 44, 29, and 19, respectively (Figure 10 and Supplementary Tables S12–S14). Among the 44 genes shared with N’Dama is *BLA-DQB*, a member of MHC class II genes and *RNF122, SERPINE3, INTS6, FAM124A, DUSP26, PIGK*, and *ST6GALNAC5*, commonly detected in Muturu and N’Dama based on *Rsb* analysis, also among the top selection signature regions in the Muturu analysis (Table 3). Additionally, seven candidate genes also belonging to the bovine MHC class II genes (*BOLA-DQA2, LOC100848815, BOLA-DQB, BOLA-DQA5, BOLA-DRB3, ENSBTAG00000003352*, and *ENSBTAG00000000432*) are commonly detected in Muturu, N’Dama and Holstein. While one gene (*PPP2R2B*) is detected in Muturu, N’Dama and Jersey only.

### Functional Annotation of Candidate Genes

To map the genes under selection in Muturu to biological processes and pathways, we performed gene enrichment (over-representation) analyses using the web-based DAVID database *v.*6.8 (Huang et al., 2009a,b). Five distinct functional annotation analyses were performed for the genes present within candidate selected regions: Muturu genes exclusively shared with N’Dama (*n* = 44), Muturu genes exclusively shared with Holstein (*n* = 29), Muturu genes exclusively shared with Jersey (*n* = 19), Muturu genes shared with N’Dama and Holstein (*n* = 7) and Muturu specific genes (*n* = 507) and the (Figure 9).

Annotation of the 44 genes shared with N’Dama indicates the importance of these genes in three candidate KEGG pathways, namely phagosome (e.g., *BLA-DQB, ATP6V0D2*, and *COLEC11*), metabolic pathways (e.g., *ATP6VOD2, ST6GALNAC5, ADI1, ALLC*, and *PIGK*) and olfactory transduction (e.g., *LOC787659, LOC507560*, and *OR6C76*). The twenty-nine Muturu candidate genes shared with Holstein are involved in innate immune response (e.g., *FBXO9, TRIM10*, and *TRIM15*), neuroactive ligand-receptor interaction (e.g., *GHR, GHRHR*, and *ADCYAP1R1*) and olfactory transduction pathways, the 19 genes shared with Jersey are involved in metabolic and arachidonic acid metabolism (e.g., *ALOX12, ALOX15, ALOX12E*, and *UGT2A1*) pathways, while the seven genes in a single common region shared between Muturu, N’Dama and Holstein are involved in adaptive immune responses. Functional annotation of the 507 unique candidate genes identified in Muturu, reveals their roles in several biological processes, including the biological processes already mentioned above, such as the olfactory transduction pathways (*n* = 31), phagosome (*n* = 5), innate immune response (*n* = 3), metabolic (*n* = 24), neuroactive ligand-receptor interaction (*n* = 5) and arachidonic acid metabolism (*n* = 3) pathways.

We also performed the enrichment analysis using the entire 607 genes under selection in Muturu. Of the numerous associated functional annotation terms revealed, six candidate enriched terms are statistically significant (FDR ≤ 0.05), these include sensory perception of smell (GO: 0007608, *n* = 25, FDR = 7.29x10^-7^), G-protein coupled receptor signaling pathway (GO:0007186, *n* = 44, FDR = 1.63 × 10-3), arachidonic acid metabolic process (GO:0019369, *n* = 6, FDR = 0.0214), Graft-versus-host disease (bta05332, *n* = 9, FDR = 0.0216), lipoxygenase pathway (GO:0019372, *n* = 5, FDR = 0.0251) and Autoimmune thyroid disease (bta05320, *n* = 10, FDR = 0.054). Nevertheless, candidate genes associated with some enriched terms that are relevant to important Muturu phenotypes, such as immunity/disease resistance (e.g., immune response, innate immune response, natural killer cell mediated cytotoxicity and phagosome), heat tolerance (e.g., protein folding), tick resistance (e.g., inflammatory response), feeding behavior (e.g., glucose homeostasis), and morphological traits (e.g., skeletal muscle fiber development) are presented in Supplementary Table S15.

### Muturu Specific Missense SNPs at Candidate Selected Regions

We identified and selected for further analyses, the non-synonymous (missense) SNPs within genes in candidate selected regions in Muturu (*iHS* and *Rsb* tests). Missense SNPs are particularly of interest because they may lead to amino acid changes in proteins, and as such may be associated with specific phenotypes, e.g., disease resistance/susceptibility. Out of the 242,910 SNPs identified within genes in Muturu, 917 are missense SNPs (0.4%). These variants were detected in 273 genes, 152 of them containing between 2 to 10 missense SNPs, and for 15 genes, including eight unannotated and seven annotated genes (*RBBP8, TEX15, LAMA3, TRIM40, LOC100848815, NID1*, and *OR12D3*), the numbers of missense SNPs ranged from 11 (*OR12D3*) to 32 (*RBBP8*) (Supplementary Table S16).

One hundred and sixty-three missense variants were found to be fixed in the 10 Muturu. Compared to the other three taurine, one SNP variant is uniquely found in Muturu (uncharacterized gene), six are uniquely found in Muturu and N’Dama, these include three located within an uncharacterized gene on BTA 12 and the remaining three within *NID1* on BTA 28 (Table 5).

**Table 5:** Genes containing fixed Muturu unique and shared missense variants.

Chr	Start	End	Gene	Allele	Frequencies			
					**MUT**	**NDM**	**HOL**	**JER**
3	67740491	67740493	*PIGK*	A	1.00	0.90	0.25	0.35
3	118280037	118280039	*TRAF3IP1*	C	1.00	0.85	0.10	0.25
3	118296231	118296233	*TRAF3IP1*	T	1.00	0.83	0.25	0.40
3	118279756	118279758	*TRAF3IP1*	A	1.00	0.80	0.10	0.20
3	118279780	118279782	*TRAF3IP1*	A	1.00	0.80	0.10	0.20
4	65801935	65801937	*GHRHR*	C	1.00	1.00	–	0.05
4	52746522	52746524	*TFEC*	A	1.00	0.20	0.35	0.40
5	27072835	27072837	*TNS2*	G	1.00	1.00	–	0.10
5	27075001	27075003	*TNS2*	C	1.00	1.00	–	0.10
5	27074317	27074319	*TNS2*	C	1.00	1.00	–	0.05
5	30986418	30986420	*DDN*	T	1.00	0.35	–	0.45
5	82655125	82655127	*Uncharacterized*	G	1.00	0.25	0.10	0.35
5	82656132	82656134	*Uncharacterized*	T	1.00	0.25	0.10	0.35
5	82656195	82656197	*Uncharacterized*	G	1.00	0.15	0.10	0.35
5	82656274	82656276	*Uncharacterized*	A	1.00	0.15	0.10	0.35
6	116025806	116025808	*CD38, PROM1*	A	1.00	0.25	0.40	0.05
8	63797826	63797828	*TBC1D2*	A	1.00	0.10	0.30	–
11	103811792	103811794	*CCDC187*	T	1.00	0.45	0.20	0.17
12	77988600	77988600	*Uncharacterized*	G	1.00	1.00	–	–
12	77988609	77988609	*Uncharacterized*	A	1.00	1.00	–	–
12	77988681	77988681	*Uncharacterized*	C	1.00	1.00	–	–
12	77988731	77988733	*Uncharacterized*	G	1.00	1.00	0.14	–
12	77988259	77988261	*Uncharacterized*	G	1.00	1.00	0.14	–
12	20858123	20858125	*SERPINE3*	C	1.00	0.95	0.25	0.30
12	74838297	74838297	*Uncharacterized*	A	1.00	–	0.33	–
12	74838323	74838323	*Uncharacterized*	C	1.00	–	0.33	–
12	74838341	74838341	*Uncharacterized*	T	1.00	–	0.33	–
13	423080	423082	*Uncharacterized*	A	1.00	0.70	0.10	–
13	424740	424742	*Uncharacterized*	C	1.00	0.65	0.10	–
13	423618	423620	*Uncharacterized*	G	1.00	0.50	0.10	–
15	81220648	81220650	*Uncharacterized*	G	1.00	0.80	0.45	0.10
15	81220574	81220576	*Uncharacterized*	T	1.00	0.70	0.45	0.15
15	81220232	81220234	*Uncharacterized*	G	1.00	0.50	0.39	0.06
15	81220249	81220251	*Uncharacterized*	G	1.00	0.50	0.13	–
17	51217737	51217739	*SRY*	A	1.00	–	0.45	0.60
17	35913849	35913849	*Uncharacterized*	T	1.00	–	–	–
19	28314450	28314452	*ALOX15B*	T	1.00	0.25	0.15	0.05
23	25405216	25405218	*BOLA-DQA5*	C	1.00	0.20	0.63	0.40
23	25405259	25405261	*BOLA-DQA5*	A	1.00	0.25	0.67	0.40
23	25405216	25405218	*BOLA-DQA5*	C	1.00	0.20	0.63	0.40
23	26354255	26354257	*Uncharacterized*	C	1.00	–	0.86	0.75
23	25351733	25351735	*BOLA-DQA2*	C	1.00	1.00	–	0.33
23	25375390	25375392	*BOLA-DQB*	T	1.00	0.33	–	0.20
23	25388099	25388099	*BOLA-DQB*	G	1.00	0.43	–	–
23	32742809	32742811	*RIPOR2*	A	1.00	0.40	0.25	0.20
23	32753726	32753728	*RIPOR2*	C	1.00	0.05	0.25	0.15
28	8798180	8798180	*NID1*	C	1.00	1.00	–	–
28	8798200	8798200	*NID1*	G	1.00	1.00	–	–
28	8798242	8798242	*NID1*	G	1.00	1.00	–	–


## Discussion

We report here for the first time the autosomal genome diversity and genome-wide candidate positive selection signatures of a West African trypanotolerant shorthorn cattle using full genome resequencing data. We compared our results with an African longhorn taurine, N’Dama and two European taurine, Holstein and Jersey. Compared to these, the Muturu population examined appears to be more genetically homogenous as translated by the lower ratio of heterozygous SNPs to non-reference homozygous SNPs (Table 1) and as illustrated by the PCA results. Most notably, none of the Muturu samples are differentiated at PC3 and PC4. It is in agreement with the known management of the breed and its endangered status. Alternatively, the relative lack of diversity may be a consequence of population bottleneck following rather environmental selection pressures. Also, our genetic distance results and proportion of SNP shared between breeds support a more recent shared ancestry between Muturu and N’Dama than between Muturu and Holstein or Jersey.

Genome-wide selection signatures were assessed in the endangered Muturu cattle in order to provide insights into and possibly unravel the genetic architecture and control of some of its important phenotypes. We have used two complementary EHH-based selection scan tests, within population *iHS* and between population *Rsb*. Both tests have substantial statistical powers to detect loci under natural selection even when sample sizes are small (Pickrell et al., 2009). In particular, *iHS* test has the power to detect partial selective sweeps within a population, while *Rsb* can detect selected alleles that have risen to near-fixation or a point of fixation in a specific population but remains polymorphic in the other population (Biswas and Akey, 2006; Voight et al., 2006). To enable us to characterize the genomic signatures of Muturu, we also carried out comparison with regions identified in three other taurine breeds following the same procedures. However, one of the limitations of this type of study is the likelihood of errors on the cattle reference genome assembly (UMD3.1) that we have utilized (Boitard et al., 2016; Utsunomiya et al., 2016; Zhou et al., 2016). Evidently, among the candidate selection signature regions identified, two regions on BTA 13 (13:5300001–5450000) and BTA 17 (17:35800001:35950000) (Table 2), have previously been reported as wrong assignments of sequences on the UMD3.1 cattle reference assembly. In particular, these regions contain *Y* chromosome specific genes, and the UMD3.1 reference assembly lacked chromosome *Y*, thus, a possible indication of the sex chromosome sequences wrongly assigned to autosomes (Boitard et al., 2016).

Although Muturu cattle and the two European breeds are both taurine, they have diverged thousands of years ago following their domestication from wild aurochs at the Near East domestication center(s) and distinct geographic continental dispersion (Loftus et al., 1999). The breeds have evolved differently, following different demographic histories, human and environmental selection pressures. In particular, the European breeds have undergone recently heavy human-mediated selection pressures for production traits such as dairy. For Muturu, natural selection, in relation to its tropical environments, rather than human selection was and remains the main selection pressure. Accordingly, it may be expected that a larger proportion of the signature of selection in Muturu may be related to environmental adaptation rather than productivity traits. Nevertheless, some candidate selected genes were identified both in Muturu and each of the European breeds, but with no overlap identified between Muturu and both European breeds (Figure 10). The majority of the 29 genes shared with Holstein (Supplementary Table S10) belongs to the olfactory receptor genes family, other genes include; *BOLA, TRIM10, TRIM15, MAP3K5, GCM1, MAP7, CHAF1B, GHRHR*, and *GHR*. The BOLA and TRIMs genes are known to have related function in adaptive and innate immune responses, respectively. Sequence variations at the growth hormone releasing hormone receptor (*GHRHR*) and the growth hormone receptor (*GHR*) have been reported to affect stature in human (Goddard et al., 1995; Ko et al., 2009; Inoue et al., 2011; Dauber et al., 2014). Polymorphisms within *GHRHR* gene have also been associated with growth traits in livestock in general such as cattle and goat (Curi et al., 2005; An et al., 2011; Liu et al., 2011). To the best of our knowledge, the *GHR* has been reported so far to be associated with milk production but not bovine stature in cattle (Blott et al., 2003; Viitala et al., 2006; Bouwman et al., 2018). However, variants in this genes have been demonstrated to affect body size in other mammalian species, such as in the case of the Laron syndrome in human (Cui et al., 2015), in miniature pig (Tian et al., 2014) and in dogs breeds (Rimbault et al., 2013; Plassais et al., 2017). Muturu cattle are particularly known for their dwarf stature, which may explain the selection signals observed in our study at the *GHR* and *GHRHR* genes. On the contrary, selection signals at the same gene regions in Holstein might be the result of selection for milk traits rather than stature. Indeed, a further investigation of the haplotype diversity in the regions of the genes reveals distinct haplotypes among the breeds (Supplementary Figure S7). Interestingly, a fixed non-synonymous T > C mutation at rs109390134 (exon 6) of the *GHRHR* gene was identified in Muturu cattle with the allele being absent in Holstein.

Muturu and N’Dama are two cattle breeds indigenous to the West Africa region. A major common phenotype is their resistance/tolerance to tropical disease such as trypanosomiasis. They are also both adapted to the humid and sub-humid tropical environments. Their trypanotolerant phenotypes have been linked to their innate capacity to control level of parasitemia and anemia (Murray and Morrison, 1978; Murray et al., 1984; Murray, 1988), processes involving innate and adaptive immune responses and hematopoietic control. Accordingly, we may expect that the shared genomic signatures of these two African breeds will point toward their distinct phenotypes.

Intriguingly, an unannotated protein coding gene, ENSBTAG00000026163, which overlapped the highest selection signal observed in Muturu on BTA 23 as revealed by the *iHS* test (Figure 4), is also shared with the N’Dama (Supplementary Tables S4, S6). Although the function of this gene in cattle is unknown, it is orthologous to the erythroblast membrane associated protein (*ERMAP*) human gene that is responsible for the recognition of the Scianna blood group (Wagner et al., 2003). Polymorphisms within the human gene have been associated with hemolytic disease in the newborn (Hue-Roye et al., 2005), the cattle orthologous as detected in our study might be of relevance to infections of blood parasites such as trypanosome species in cattle.

Among other genes overlapping candidate selected regions on BTA 23 in the two West African taurine breeds are members of the MHC class II genes. In cattle and other ruminant species, the MHC region is highly polymorphic and is a major source of variation in immune responses. The bovine MHC, also known as BoLA (bovine leucocyte antigen) complex, is of particular interest to animal breeders and veterinary geneticists because of its links to genetic resistance and susceptibility to several diseases. For instance, polymorphisms in *BOLA-DRB3* has been linked to resistance to bovine leukemia virus (BLV) infection (Xu et al., 1993; Amills et al., 1998). Some other studies have linked it with tolerance/resistance to several cattle diseases such as mastitis, tick-borne infection and tick-borne pathogen infections (Sharif et al., 1998; Martinez et al., 2006; Kulberg et al., 2007; Rupp et al., 2007; Duangjinda et al., 2013). Other evolutionary factors such as genetic drift and population bottlenecks may have also played a role in the diversity at the MHC loci (Mikko and Anderson, 1995; Amills et al., 1998; Lewin et al., 1999). We may, therefore, hypothesize that common phenotypes in Muturu and N’Dama such as trypanotolerance may be associated with this variation within the MHC gene region. Accordingly, a fixed non-synonymous (missense) variant, T > C mutation (rs208515389) at exon 3 of the *BOLA-DQA2* gene, is found both in Muturu and N’Dama. The allele is absent in Holstein and at a low frequency (30%) in Jersey. Further investigation of this MHC gene region between trypanotolerant and susceptible cattle populations will be necessary to further support a possible association between this polymorphism and the trypanotolerance of the two breeds. Another gene related to parasitic disease challenges and found in both breeds, is *DUSP26*. This gene has been identified among highly expressed genes in resistant group of goat exposed to gastrointestinal nematode (GIN) infections (Bhuiyan et al., 2017), making it an interesting candidate for further investigation in indigenous African taurine cattle.

Heat stress is also a common environmental challenge for the two breeds. Here, a gene of interest commonly identified at a candidate selected region in both African taurine is *INTS6*. Although, both the African taurine share similar haplotype at the gene region with the European taurine, the haplotype frequencies is nearly fixed in the African taurine but at a low and moderate frequencies in the Holstein and Jersey, respectively (Supplementary Figure S7). The *INTS6* gene has been shown to be upregulated in peripheral blood leukocytes of cattle and buffalo exposed to heat stress (Kolli, 2012). The gene has also been showed to be upregulated following exposure to UV irradiation, with a switch from the expression of long mRNA isoforms to short isoforms and preferential use of short alternative last exon (ALE) transcript isoforms (Williamson et al., 2017). The signature of selection signal in the genomic region overlapping *INTS6* may be therefore the results of the climatic selection pressures (high temperature and UV radiation), witnessed by these two taurine cattle living in tropical areas.

Eighty three percent (*n* = 507) of the genes in candidate selected regions in Muturu are not detected under positive selection in any of the three *B. taurus* breeds studied. Among the Muturu unique candidate genes, *TFEC* and *OSBPS* were the only two genes identified in the *Rsb* comparisons of Muturu with each of the other breeds (Figure 8). Of particular interest is the transcription factor *TFEC*, a gene found within a candidate selected region on chromosome 4 (*iHS* analysis). It contains a missense SNP (G > A, gCt/gTt) that is fixed in Muturu (Table 5). *TFEC* is a member of the *MIT-TFE* gene family of transcription factors which includes *MITF, TFE3*, and *TFEB* (Hemesath et al., 1994). *TFEC* is mainly expressed in cells of myeloid origin, *MITF* is predominantly expressed in melanocytes, osteoclasts, mast cells, macrophages, NK cell, B cells and heart, while *TFE3* and *TFEB* are thought to be more-ubiquitously expressed (Rehli et al., 1999; Martina et al., 2014). Homo-dimerization and hetero-dimerization within members of the MITF/TFE family are critical for the binding to DNA and the transcriptional activation of the target genes (Martina et al., 2014). The microphthalmia-associated transcription factor (*MITF*) is involved in the development of melanocytes and melanoma and to regulate transcription of a broad range of genes, ranging from genes important for pigment production to genes involved in cell cycle regulation, migration and survival. When *TFEC* acts like an isoform of *MITF* it is involved in retinal pigment epithelium (RPE) differentiation (Hemesath et al., 1994; Levy et al., 2006; Bharti et al., 2012; Raviv et al., 2014). Another gene within a Muturu specific candidate selected genome region (*iHS* analysis) is *KIT*. It is known to interact with *MITF*, which also plays an important roles in melanogenesis (Besmer et al., 1993). Selection signatures spanning the regions of both the *KIT* and the *MITF* genes have been associated with coat color variations in several animals species including pigs (Rubin et al., 2012), sheep (Fariello et al., 2014), goats (Benjelloun et al., 2015; Wang et al., 2016; Guo et al., 2018), horses (Hauswirth et al., 2012, 2013), and cattle (Fontanesi et al., 2010; Hayes et al., 2010; Whitacre, 2014; Kim et al., 2017). These two genes and their interaction in Muturu cattle may explain the different coat color patterns also observed in Nigerian Muturu (Adebambo, 2001) including the white spotting in Holstein (Liu et al., 2009). Interestingly, both Muturu and Holstein share similar haplotype at high frequencies at the *KIT* gene region, compared to the other two taurine breeds (Jersey and N’Dama) in which they segregate for a number of different haplotypes at the same region (Supplementary Figure S7).

Other identified Muturu specific genes include *ZRANB3* and *MAP3K5* related to feed efficiency/residual feed intake in cattle and pigs (Bovine HapMap Consortium, 2009; Do et al., 2014; Taye et al., 2017), *ABCG2* and *LAP3* related to milk production (Blott et al., 2003; Cohen-Zinder et al., 2005; Ron et al., 2006; Flori et al., 2009), *RFX2, SRY, LAP3*, and *GPX5* related to reproduction and fertility (Horvath et al., 2004; Szreder and Zwierzchowski, 2004; Chabory et al., 2010; Mishra et al., 2013; Braud et al., 2017), *ACTA1* and *CTNNB1* related to heat stress (Kecskés et al., 2015; Guo et al., 2016), and *PRKAG3* related to meat quality traits in domestic cattle and pigs (Reardon et al., 2010; Ryan et al., 2012; Zhang et al., 2015; Bongiorni et al., 2016). The later gene has also recently been reported as a cattle candidate domestication gene due to its extensive miRNA binding site polymorphisms between *B. taurus* and *B. primigenius* (Braud et al., 2017).

A number of unique Muturu candidate regions and genes was detected following the *Rsb* comparison between Muturu and N’Dama. Among them are the *GFI1B, NPC1, RCAN1*, and *OLA1* genes, for which the two African taurine breeds have distinct haplotypes in the gene regions (Supplementary Figure S8). These genes may therefore, provide an insight into selected differentiate regions between West African shorthorn and longhorn taurine population. *GFI1B* is essential for neutrophil differentiation and it is required for the development of both erythroid and megakaryocytic lineages. Congenital mutations of the gene in human and mouse have been related to abnormal platelet function and decrease in erythropoiesis of embryonic stem cells, respectively (Karsunky et al., 2002; Saleque et al., 2002; Anguita et al., 2016). *NPC1* has been reported to play an important role in subcellular lipid transport, homeostasis, platelet function and formation. Louwette et al. (2012) reported that *NPC1* defect resulted in abnormal platelet formation and function which imply that *NPC1* gene may show a role in platelet function and formation. Other studies have reported the roles of *NPC1* in the control of appetite in mice (Xie et al., 1999), obesity in human (Meyre et al., 2009; Mejía-Benítez et al., 2013) and in possible regulation of bovine growth and body development (Dang et al., 2014). *OLA1* (Obg-like ATPase 1) has important roles in the regulation of cellular stress responses such as oxidative stress (Zhang et al., 2009) and improved thermal resistance in mammalian cells (Mao et al., 2013). The latter role is mediated by the gene involvement in the stabilization and subsequent upregulation of the heat shock *HSP70* gene (Mao et al., 2013).

The aforementioned important functions of *GFI1B, NPC1*, and *OLA1* are indicative of their importance in Muturu adaptation to the tropics. The influence of *GFI1B* and *NPC1* on erythropoiesis and feed intake, respectively, could be related to the trypanotolerance of Muturu, while *OLA1* involvement in the thermotolerance of Muturu is possible. However, both Muturu and N’Dama are known for their tolerance to trypanosomiasis and they both have intrinsic thermotolerance. Therefore, these genes may represent example of genes contributing to common phenotypes but under positive selection in one breed and not the other.

More so, three unique Muturu candidate genes, namely *FAS, IDO2*, and *PLCB1*, are part of the candidate African trypanosomiasis KEGG pathway (BTA05143) revealed following functional annotation terms by DAVID bioinformatic tool (Supplementary Table S15). Generally, our results indicate some of the enriched biological processes and associated candidate genes in Muturu may have important roles in their adaptation phenotypes. However, a transcriptomic analysis may provide further support for the importance of these pathways including tolerance of Muturu to trypanosome infection in cattle.

Interestingly, we do find in Muturu, for a few genes, a large number of missense mutations, the majority of them novel. This will need to be confirmed through the analysis of more samples and targeted resequencing. It indicates the presence of outlier haplotypes in the genome of Muturu cattle within candidate selected regions. Investigating their origins will be of interest in the ongoing debate of possible presence of African auroch *Bos primigenius* introgression in the today genome of African cattle (Decker et al., 2014).

## Conclusion

The work reported here is the first full autosomal genome-wide scan which aims to unravel genomic selection signatures in a trypanotolerant West African shorthorn Muturu using whole genome re-sequencing. Population structure analysis revealed clearly genetic differences between African and European *B. taurus*, and between the African *B. taurus* (Muturu and N’Dama). Selection signature analyses based on *iHS* and *Rsb* revealed several loci under selection in Muturu and the three other *B. taurus* breeds studied. The majority of the identified candidate genes are unique to each breed, while the few overlaps are genes with related functions in immune responses. The shared selection signatures between the two West African *B. taurus* could be related to their adaptation to local environmental, particularly their trypanotolerance traits. This result could be a pointer for further investigation into the genetic basis of trypanotolerance in the entire West African *B. taurus* population. However, additional studies such as comparative genomics analyses between a larger group of trypanotolerant and trypanosusceptible cattle populations may be required to further confirm the results obtained here. More so, across the Muturu genome and in some of the identified selection signature regions are several unannotated regions (gene desert regions) which may limit our full understanding of the genetic mechanisms of adaptation and other important phenotypes/traits in this population.

## Author Contributions

AT, OH, and ON conceived the project. AT and OH designed the experiment. ON and AE provided the support during sampling. AT performed the analyses. YU provided the bioinformatics support. AT prepared and wrote the manuscript. YU and OH revised the manuscript. All authors read and approved the final manuscript.

## Conflict of Interest Statement

The authors declare that the research was conducted in the absence of any commercial or financial relationships that could be construed as a potential conflict of interest.

## Footnotes

1 https://www.bioinformatics.babraham.ac.uk/projects/fastqc/

2 https://software.broadinstitute.org/gatk/best-practices/

3 http://broadinstitute.github.io/picard

4 ftp://ftp.ncbi.nih.gov/snp/organisms/archive/cow_9913/chr_rpts/

5 http://www.ensembl.org/biomart

6 http://david.abcc.ncifcrf.gov/

7 https://forge-dga.jouy.inra.fr/projects/hapflk
