# Supplementary material for: Genome Sequence Analysis Reveals Selection Signatures in Endangered Trypanotolerant West African Muturu Cattle
Source: Front Genet. 2019 Jun 4;10:442. doi: 10.3389/fgene.2019.00442 (PMC6558954; doi:10.3389/fgene.2019.00442)
Supplement: Supplementary file 2 [file Data_Sheet_2.pdf]

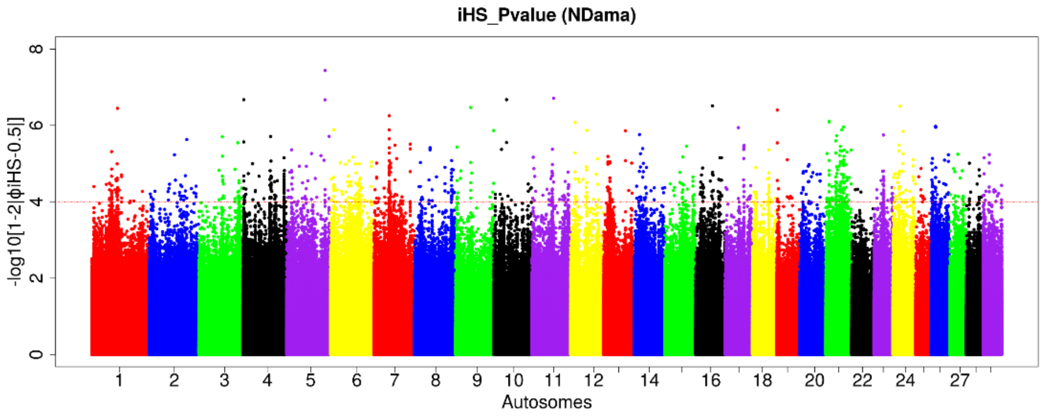

Supplementary Figure S1 | Manhattan plot of the genome-wide distribution of *iHS* scores for the autosomes in N'Dama cattle. The red dash line indicates the threshold of P < 0.0001 (equivalent of –log (*iHS*) > 4) at which windows are considered under selection

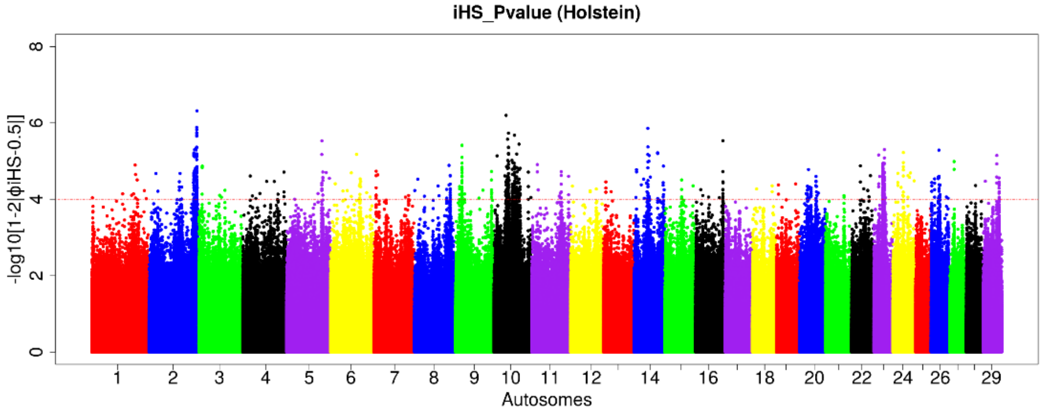

Supplementary Figure S2 | Manhattan plot of the genome-wide distribution of *iHS* scores for the autosomes in Holstein cattle. The red dash line indicates the threshold of P < 0.0001 (equivalent of –log (*iHS*) > 4) at which windows are considered under selection

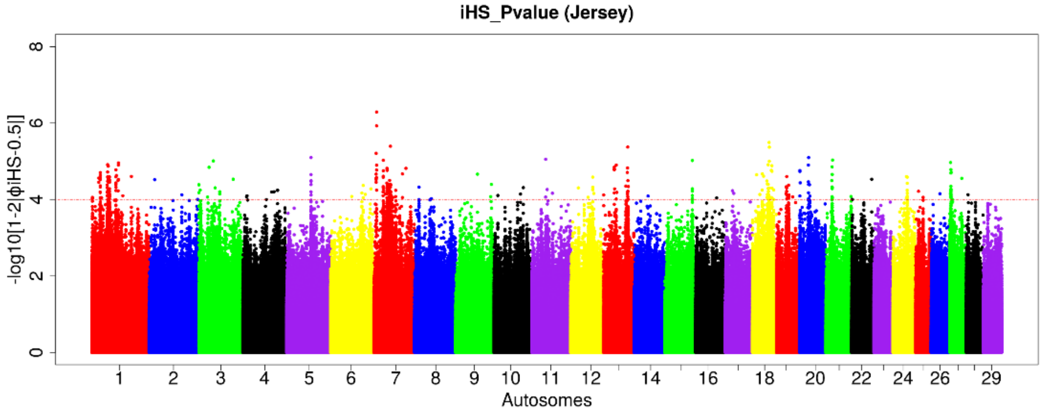

Supplementary Figure S3 | Manhattan plot of the genome-wide distribution of *iHS* scores for the autosomes in Jersey cattle. The red dash line indicates the threshold of P < 0.0001 (equivalent of –log (*iHS*) > 4) at which windows are considered under selection

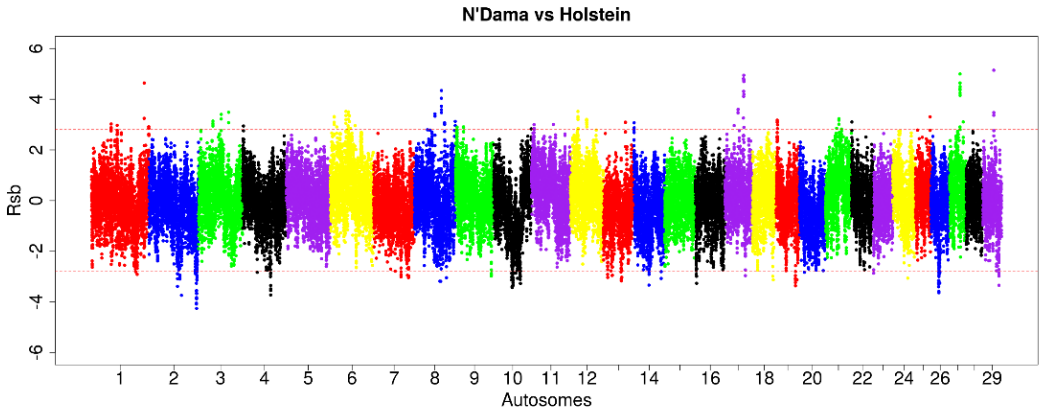

Supplementary Figure S4 | Manhattan plot of the genome-wide distribution of *Rsb* N'Dama - Holstein. Red dash lines are the threshold of the windows in the top 0.5% *Rsb* scores

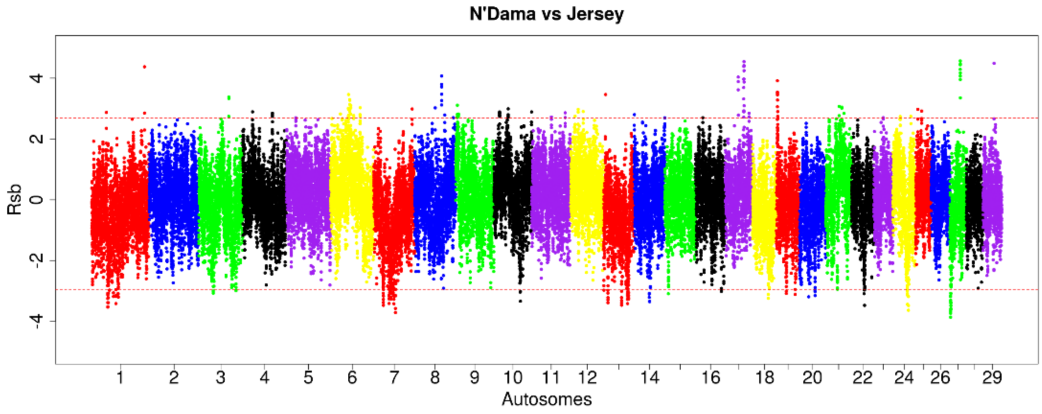

Supplementary Figure S5 | Manhattan plot of the genome-wide distribution of *Rsb* N'Dama - Jersey. Red dash lines are the threshold of the windows in the top 0.5% *Rsb* scores

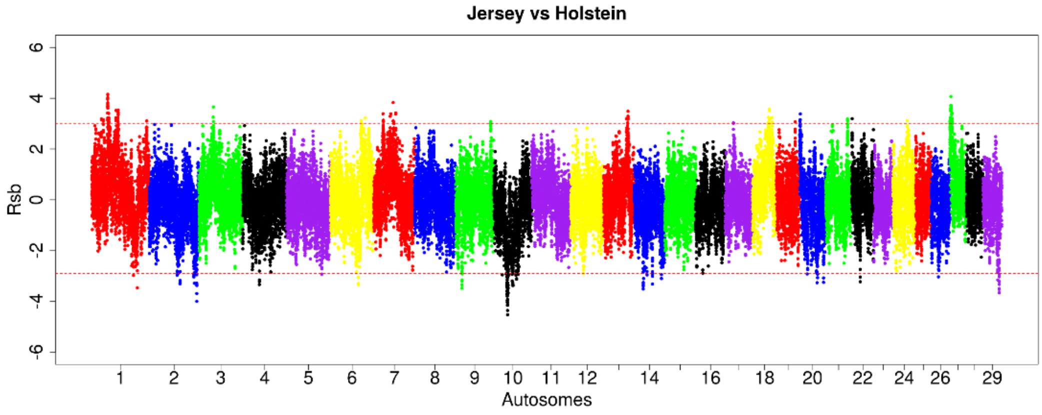

Supplementary Figure S6 | Manhattan plot of the genome-wide distribution of *Rsb* Jersey - Holstein. Red dash lines are the threshold of the windows in the top 0.5% *Rsb* scores

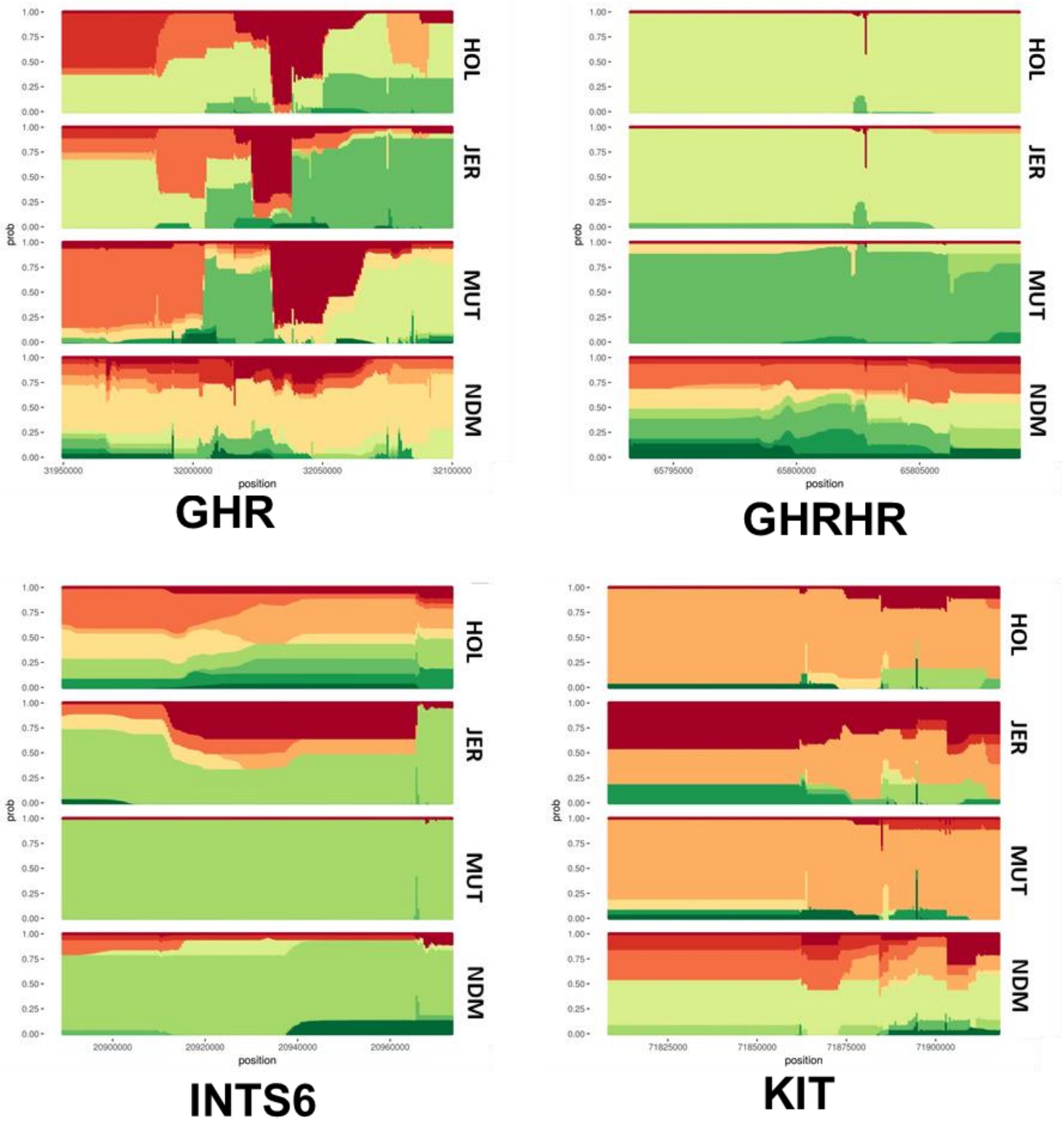

Supplementary Figure S7 | Haplotype diversity for African and European taurine breeds in genomic regions of four candidate genes between Muturu and other taurine breeds

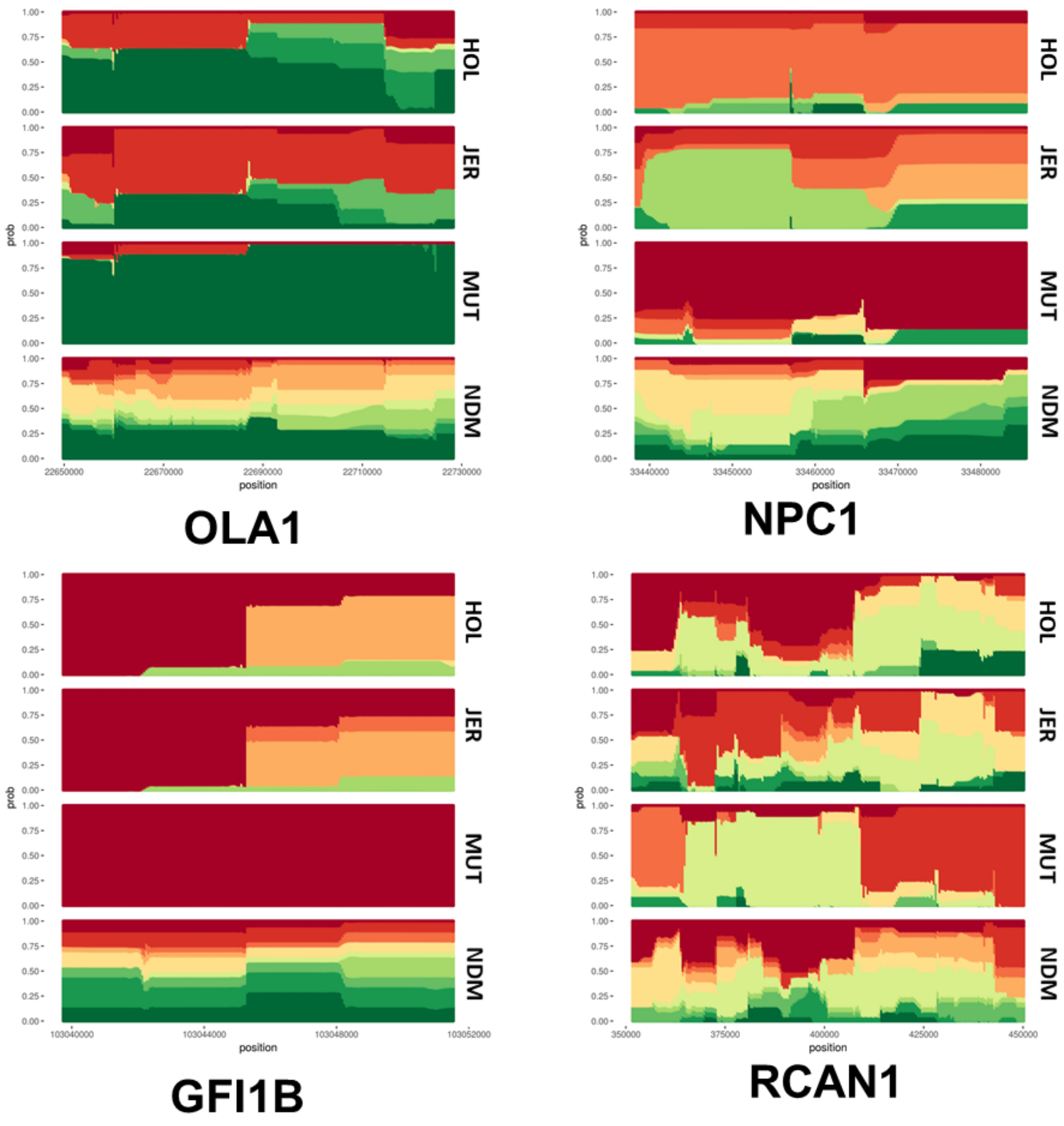

Supplementary Figure S8 | Haplotype diversity for four taurine breeds in genomic regions of four Muturu candidate genes detected following Rsb comparison test between Muturu and three other *Bos taurus* breeds.
